# Identification of Complex Health Interventions Suitable for Evaluation: Development and Validation of the 8-Step Scoping Framework

**DOI:** 10.2196/10075

**Published:** 2019-03-05

**Authors:** Rosemary Davidson, Gurch Randhawa, Stephanie Cash

**Affiliations:** 1 Institute for Health Research University of Bedfordshire Luton United Kingdom; 2 Flying Start Luton United Kingdom

**Keywords:** complex interventions, early years, evaluation, multistakeholder provision

## Abstract

**Background:**

There is extensive literature on the methodology of evaluation research and the development and evaluation of complex interventions but little guidance on the formative stages before evaluation and how to work with partner organizations that wish to have their provision evaluated. It is important to be able to identify suitable projects for evaluation from a range of provision and describe the steps required, often with academic institutions working in partnership with external organizations, in order to set up an evaluation. However, research evaluating programs or interventions rarely discusses these stages.

**Objective:**

This study aimed to extend work on evaluability assessment and pre-evaluation planning by proposing an 8-Step Scoping Framework to enable the appraisal of multiple programs in order to identify interventions suitable for evaluation. We aimed to add to the literature on evaluability assessment and more recent evaluation guidance by describing the processes involved in working with partner organizations.

**Methods:**

This paper documents the steps required to identify multiple complex interventions suitable for process and outcome evaluation. The steps were developed using an iterative approach by working alongside staff in a local government organization, to build an evidence base to demonstrate which interventions improve children’s outcomes. The process of identifying suitable programs for evaluation, thereby establishing the pre-evaluation steps, was tested using all Flying Start provision.

**Results:**

The 8-Step Scoping Framework was described using the example of the local government organization Flying Start to illustrate how each step contributes to finding projects suitable for process and outcome evaluation: (1) formulating overarching key questions that encompass all programs offered by an organization, (2) gaining an in-depth understanding of the work and provision of an organization and engaging staff, (3) completing a data template per project/program offered, (4) assessing the robustness/validity of data across all programs, (5) deciding on projects suitable for evaluation and those requiring additional data, (6) negotiating with chosen project leads, both within and outside the organization, (7) developing individual project evaluation protocols, and (8) applying for ethical approval from the university and partner organization.

**Conclusions:**

This paper describes the processes involved in identifying suitable projects for evaluation. It adds to the existing literature on the assessment of specific programs suitable for evaluation and guidance for conducting evaluations by establishing the formative steps required to identify suitable programs from a range of provision. This scoping framework particularly relates to academic partners and organizations tasked with delivering evidence-based services designed to meet local needs. The steps identified have been described in the context of early years provision but can be applied to a range of community-based evaluations, or more generally, to cases where an academic partner is working with external stakeholders to identify projects suitable for academic evaluation.

## Introduction

There is extensive literature on evaluation research methodology and development and evaluation of complex interventions, from identifying existing evidence to measuring outcomes and understanding processes [1-7]. However, there is little guidance on the formative stages of identifying suitable services/programs for evaluation and the ways to work with partner organizations that wish to have their provision evaluated in order to build an evidence base related to their particular local, geographical or cultural context beyond basic advice [8-10]. A possible disadvantage of conducting a robust evaluation is the risk of finding no change or negative results, potentially influencing future funding decisions and reputations.

Research on program evaluation rarely discusses the steps involved prior to evaluation in order to identify suitable projects, often with academic institutions working in partnership with external organizations to set up an evaluation. Guidance assumes that projects have already been identified, providing detailed instructions to plan and conduct evaluations. For example, Newcomer et al assumed that the projects to be evaluated were already chosen, and evaluators and organization staff had planned their evaluation approach [11]. They described the fundamental considerations that evaluators and organizations should address before beginning any evaluation activities, starting with matching evaluation approach to key questions, producing methodological rigor and appropriate evaluation design, or identifying ways to apply an evaluation framework in a particular disciplinary context [12].

Pre-evaluation activities are mostly discussed in the literature on evaluability assessment, which was first conceptualized in the late 70s [13] after the costly, large-scale evaluations of major social interventions in the United States in that period reported no benefit. Poor evaluation approaches and ultimately, disappointing results, were thought to be the result of inadequate program definition and lack of development and specification of causal links between intervention actions and expected results. In response, a “pre-assessment of evaluability” was developed to improve evaluation methodology, not by assessing whether a program can be evaluated, “but [by] whether the program is ready to be managed to achieve desired performance and outcomes, what changes are needed to allow results-oriented management, and whether evaluation is likely to contribute to improved program performance” [14]. Evaluability assessment has been revived in recent years, because the demand for evidence-based practice of has increased [15].

Evaluability assessment is a systematic method to plan robust evaluations as well as a “low-cost pre-evaluation activity to prepare better for conventional evaluations of programmes” [16] in order to make sound decisions on evaluation methodology before funds are committed. The approach is viewed as a way to balance the growing demand for evidence through evaluation when limited resources are available [15]. A recent rapid scoping review showed the range of interventions that have been assessed by evaluability assessment methodology to determine their suitability for evaluation, such as the State Asthma Programme [17], the Healthy Community Challenge Fund [18], and National Driver Retraining Programme [19].

Evaluability assessment focuses on the feasibility of evaluating a specific intervention and usually involves the following key stages: structured engagement with stakeholders to understand the context of a particular intervention and ensure evaluation findings are meaningful, development of a theory of change to inform implementation and identify key outcomes, review of existing literature and data to establish quality of evidence already available, and recommendations for proposed evaluation designs. Evaluability assessment allows researchers to assess the suitability of a specific intervention for evaluation by working through the aforementioned four stages.

As it is designed to assess the suitability of a particular intervention for evaluation, it assumes that organizations that want to have their provision evaluated have the expertise to identify projects suitable for evaluation. The 8-Step Scoping Framework detailed here guides researchers and stakeholders through the stages prior to evaluability assessment, where the appetite for evaluation exists but the scope is ill defined. The 8 steps described are discussed using an example of a local government organization, Flying Start, to illustrate how each step contributes to the ultimate aim of identifying projects suitable for a process and outcome evaluation.

Flying Start [20] is part of Luton Borough Council, which is a part of the UK local government. It is a unitary local authority; as such, it provides all local services including health and social care, education, and learning. Flying Start aims to improve social, emotional, and health outcomes for children from the point of pregnancy to the age of 5 years. The importance of the early years and inequality in developmental outcomes is well documented [21-26]. Flying Start and the University of Bedfordshire are developing a process and outcome-evaluation framework to establish the efficacy of their multistakeholder provision, find evidence of what works, and ensure the provision offered meets local needs [27].

In this paper, the term “provision” most often refers to all the work of an organization to discern what it offers; “programs” or “services” are terms more likely used by an organization to describe the services they offer to the public or clients; and “intervention” is the more scientific term researchers favor to describe a project, program, or service that is subject to a process and outcome evaluation.

There is limited information in the literature about the steps required to identify suitable interventions before conducting an evaluation. This paper therefore aims (1) to extend work on evaluability assessment and pre-evaluation scoping by proposing an 8-Step Scoping Framework to be applied prior to evaluability assessment to enable the appraisal of multiple programs in order to identify interventions suitable for evaluation and (2) to add to the literature on evaluability assessment and more recent evaluation guidance by describing the processes involved when working with partner organizations.

## Methods

**Figure 1:**
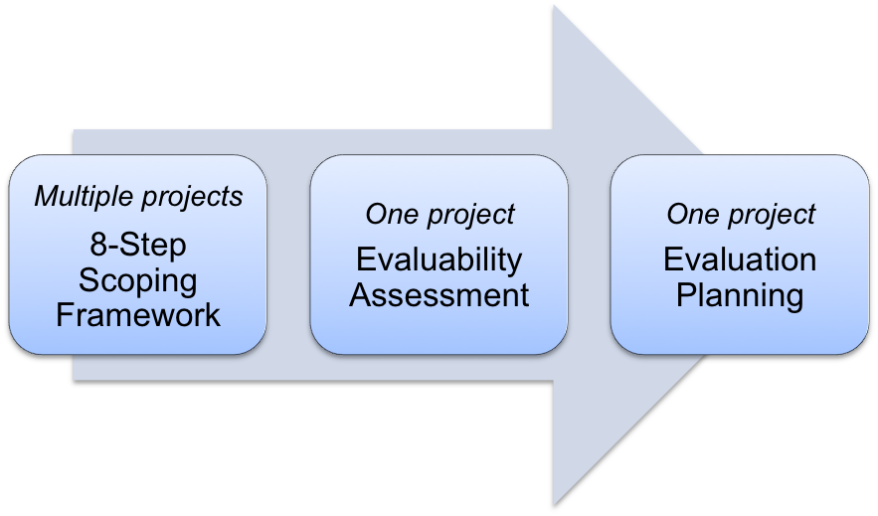
Context of 8-Step Scoping Framework in relation to evaluability assessment and evaluation planning.

This paper documents the steps involved in identifying multiple complex interventions suitable for process and outcome evaluations. We developed an 8-Step Scoping Framework to identify complex health interventions. The framework guides the selection of suitable interventions for evaluation from a range of projects. Evaluability assessment allows in-depth appraisal of one project and is particularly important when considering the evaluation of larger, costly interventions before making a decision to commission an evaluation and begin detailed evaluation planning (Figure 1).

To refine these stages, an iterative approach was taken in order to develop the steps described below. The process was developed through regular meetings between an academic institution and stakeholders, which allowed a collaborative and reflexive process where researchers reported progress and were able to form a critical understanding of stakeholder priorities, and practical considerations were balanced with research objectives. A log was maintained to document progress during the process of identifying projects suitable for evaluation.

The 8-Step Scoping Framework was developed over an 11-month period through a series of meetings with Flying Start, Luton Borough Council, and associated stakeholders. Table 1 details the nature of the meetings, attendee numbers, affiliations, and their roles in the development of the pre-evaluation framework. The meetings were conducted concurrently during the development of the framework. All meetings, except the Scoping Framework planning meetings, were ongoing as part of Flying Start’s activities. The Flying Start staff meetings allowed researchers to gain an in-depth understanding of Flying Start provision, range, number, and development of services. The Partnership Board and Project Evaluation Group meetings allowed the input of a range of professionals on evaluation scoping strategy, identification of framework steps, and feedback on framework development through various iterations. The Scoping Framework-planning meetings were devoted to reporting framework progress and allowed researchers to apply the developing scoping criteria to Flying Start projects.

Formulation of the steps was led by the following key aims: to determine ways to obtain a full understanding of the provision offered by an organization; to arrive at a consensus on the type of questions to be asked in order to assess suitability of provision for evaluation; to find the best way to obtain such information from stakeholders; to refine the scoping process to allow a decision on suitable projects, space for negotiation with project leads, and development of stand-alone project-evaluation protocols per chosen project.

Meeting minutes with decisions made were typed up and circulated for comment and discussion as the framework steps were defined and clarified. The process of identifying suitable programs for evaluation, thereby establishing the 8 scoping steps, was tested using all Flying Start provision. During the framework-development period, 36 programs/services were offered by Flying Start to families in Luton. First and successive drafts of the Scoping Framework were presented to Flying Start, council staff, researchers, and associated stakeholders over time.

**Table 1:** Characteristics of the meetings used to develop the 8-Step Scoping Framework.

Characteristic	LBC^a^ and Flying Start Partnership Board	Flying Start Project-Evaluation Group	Flying Start staff	Scoping Framework planning
Number of meetings held over the framework-development period	5 (bimonthly)	8 (every 4-5 weeks)	10 (monthly)	16 (every 2-3 weeks)
Purpose	Multiagency meeting to discuss issues related to early years services	Forum to discuss evaluation approaches for Flying Start services	Staff to update on progress and discuss any arising matters	Mapping of Scoping Framework progress
Attendees	Council heads of services, Flying Start staff, early years and public sector organizations, midwives, nutritionists, general practitioners, and councilors	Flying Start staff, LBC staff, and University of Bedfordshire staff	Flying Start staff, practitioners working in early years services, and university evaluation team	Flying Start senior staff and university evaluation team
Approximate number of attendees	15-20 stakeholders	8-12	15-20	3-5
Role in framework development	Input from a range of professionals and feedback on framework development	Identifying framework steps through various iterations	Gaining in-depth understanding of Flying Start provision, range, and number of services	Applying scoping criteria to all Flying Start services/programs offered

^a^LBC: Luton Borough Council.

## Results

**Figure 2:**
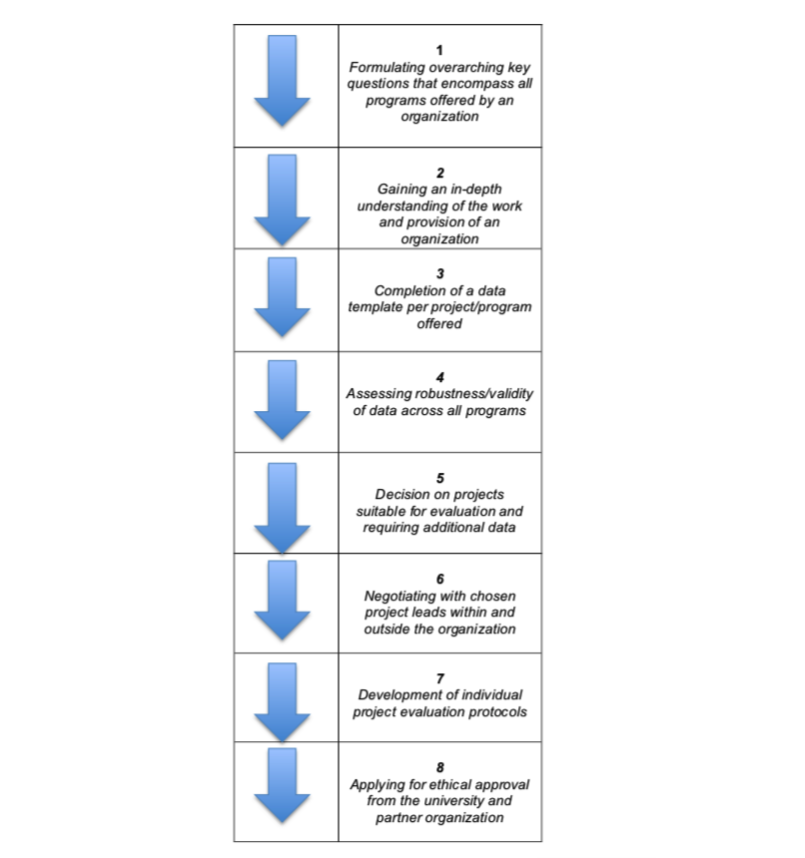
The 8-Step Scoping Framework for identifying complex health interventions suitable for evaluation.

The steps in the 8-Step Scoping Framework are presented in Figure 2. The steps are discussed using a specific example of the local government organization Flying Start to illustrate how each step contributes to the ultimate aim of finding projects suitable for process and outcome evaluation.

### Step 1 – Formulating Overarching Key Questions That Encompass All Programs Offered by an Organization

This may sound like an obvious first step, but it is important to determine whether the organization has an overarching aim guiding the content and purpose of their provision. Can this be translated into a research question to guide an evaluation? When considering multiple projects, is there a coherent research question that encompasses all projects? Most organizations will have key aims or a mission statement that can be reframed as a research question, which serves as a useful guide to ensure the overall evaluation strategy retains its focus and that the research question aligns with the objectives of the organization. In the case of Flying Start, a part of the Luton Borough Council, the overarching research question to guide the evaluation strategy was “What impact does an integrated early year’s strategy make on a life ready for learning at age five in a unitary authority?” This question was divided further into three subquestions:

- Has Flying Start succeeded in improving child/family outcomes?
- Was Flying Start more successful with certain groups and why?
- What aspects of Flying Start did participants (families) find most beneficial?

Irrespective of whether the organization has specific programs it wishes to evaluate or is led in consultation with an academic partner, the next step is vital.

### Step 2 – Gaining an In-Depth Understanding of the Work and Provision of an Organization

In the case of Flying Start, the requirement to evaluate their provision was a priority, but what was to be evaluated was unclear. Through a series of meetings, a logic model was developed to identify a set of questions that would help to both understand the provision and assess its suitability for evaluation [28]. What services would lend themselves to a robust process and outcome evaluation, which could then be published in peer-reviewed academic journals, thereby building a credible evidence base for their work? How to be strategic with the evaluation, given the finite resources and research capacity? In order to answer these questions, it is essential to negotiate access to the organization and be available to attend meetings, particularly where staff are given a forum to discuss their work and current progress. What may seem to be one organization from the outset is, in fact, a complex structure consisting of staff working in a considerable range of ways to deliver services. The researcher can begin understanding in detail how provision fits together, who it is aimed at, and the level of need it attempts to address.

Underlying all these issues, however, is an understanding of the pressures an organization faces, such as lack of staff, limited resources, responding to diverse and changing needs of a community, government guidelines, policy steers with ebbs and flows in funding, getting services up and running, and reaching those who need help most but are least likely to access services. Attending meetings and building relationships cement trust [1] and allays fears around being the subject of an evaluation. If a researcher is available to answer questions and give advice more generally about research and evaluation, it is possible to be a valuable resource to the organization in terms of embedding evaluation methodology across all services, not only the interventions chosen for evaluation.

### Step 3 – Completion of a Data Template per Project/Program Offered

Understanding the range and content of an organization’s provision is different from establishing suitability for evaluation. With Flying Start, a template was developed for project/service leads to complete requesting information on target audience for service; service aims; whether any baseline data was collected prior to inception; key performance indicators linked to outcomes (eg, communication and language, nutrition and diet, and social and emotional development); the data available and what it signifies; who owns the data (is it in the public domain?); who the data custodian is; how often data is collected; opportunities for tracking cohorts, size, and scale of the intervention; coverage (population, town wide, or ward level); whether participants (parents/families) are likely to be involved in more than one program; and the start date and length of intervention (Multimedia Appendix 1).

Researchers also agreed on key terminology during the process to avoid misunderstandings. For example, the term “provision” was used to describe the range of work an organization does to discern what was offered; “programs” or “services” were used to describe what was offered to the public or clients; and “intervention” was the scientific term used to describe a project, program, or service that lends itself to a process and outcome evaluation.

An evaluation workshop was then organized with all project leads and other key Flying Start staff with the aim of encouraging staff to think about ways to evaluate their provision, answer evaluation and research questions, and provide guidance on how to fill in the data templates. A total of 36 programs/ services were offered by Flying Start to families in Luton. The staff divided them into 8 domains: Antenatal/baby, Communication, Life course approach to healthy weight, Child mental health, Strong and supportive parents, System changes, Child safety, and Other. The event helped engage stakeholders in meaningful ways [29], foster partnerships, agree on the remit of different programs [6], support staff on ways to build an evidence base demonstrating the efficacy of their provision, and offer overall criteria to refer to in order to understand why some provision may be more suitable for evaluation. Possession of the completed data templates allowed different projects to be assessed and compared, leading to the next step.

### Step 4 – Assessing Robustness/Validity of Data Across All Programs

With the completed data templates, the task of assessing suitability of projects for evaluation could be approached systematically. It was easier to approach project leads with any follow-up questions after referring to the templates. Key criteria (Table 2) for deciding on projects were assessment of quality of data (ie, use of validated outcome tools/scales for data collection); presence of an explicit statement on the causal assumptions of how the intervention will work [1]; presence of a robust existing theory underpinning the intervention [29]; whether a theory can be identified or developed if no theory is evident [2]; presence of any other factors that drive the program, such as experience and professional practice [1]; whether the intervention is developed to a point that it can reasonably be expected to have a worthwhile effect [2]; whether there are systems or protocols already built in to projects to collect the process and outcome data required; and whether it is possible to make comparisons with control groups in order to measure progress of those using a service or participating in an intervention. Practical considerations were paramount; for example, had a project already started [9]? How long would the project/intervention run for? What were the funding restrictions? Strategically, a project may appear to be on a smaller scale, but may run for a sufficient amount of time to produce multiple cohorts of participants and therefore yield the quantity and depth of data required.

Services that appear suitable for evaluation on first inspection may, in fact, be in the early stages of implementation or facing implementation problems such as issues with recruitment or referral processes. Such problems are particularly significant when working with vulnerable families to, for example, assess the level of support required and willingness to engage with or attend services. Strategic decisions may have to be made to focus attention and resources elsewhere if an otherwise suitable program is facing problems with, for example, implementation, recruitment, or referrals of suitable participants. An exception may be made if a decision is taken to focus only on an early stage process study of an intervention that may not take off but may have strategic importance to an organization and contribute to academic debate. Such negotiations are most constructive when the preceding steps have been followed, allowing for face-to-face discussions and fruitful working relationships.

### Step 5 – Decision on Projects Suitable for Evaluation and Requiring Additional Data

By applying the abovementioned criteria in step 4, the projects lending themselves to evaluation were identified. Research capacity [1] was then used as a guiding factor to ascertain what was possible to take on, by producing an evaluation timetable with timelines for each project under evaluation. As Flying Start offers a wide range of early years provision, it was also important, where practicably possible, to reflect diversity in the projects chosen. The provision/ interventions chosen were Sign 4 Little Talkers/Big Feelings [30,31], which uses sign language to support the development of language, vocabulary, and positive behavior in children below 5 years of age; Healthy Exercise Nutrition for the Really Young (HENRY) [32-34], an obesity-prevention program for families with children below 5 years of age; Incredible Years [35-37], a parenting program for high risk socioeconomically disadvantaged families with children aged 3-5 years old having behavioral problems; and Parents as Partners [38,39], which offers counselling to improve couple relationships in order to improve child well-being and developmental outcomes (Table 3).

**Table 2:** Criteria for assessing data related to projects/interventions.

Criteria	Sign 4	HENRY^a^	Incredible Years	Parents as Partners
Data quality/outcome data	Sufficient	Sufficient	Sufficient	Sufficient
Theoretical basis	Yes	Yes	Yes	Yes
In-built evaluation tools	Yes	Yes	Yes	Yes
Prior evidence of positive effect	Pilot data	Yes	Yes	Yes
Control group comparisons	Retrospective and baseline data	Baseline data	Baseline data	Baseline data
Has the project started?	Yes	Yes	Yes	Under negotiation
Funding terms	Funded	Funded	Funded	Under negotiation
Is it scalable or does it involve multiple cohorts?	Yes	Yes	Yes	Yes

^a^HENRY: Healthy Exercise Nutrition for the Really Young.

**Table 3:** Complex interventions identified by application of the 8-Step Scoping Framework.

Project characteristics	Sign 4	HENRY^a^	Incredible Years	Parents as Partners
Scope	To improve vocabulary and communication in preschool children	Obesity prevention for parents of preschool children	To address early onset behavioral problems in preschool children	To improve couple relationship quality impacting children’s outcomes
Aims	To investigate the impact of Sign 4 on early years outcomes and implementation, and lay and professional views	To investigate pre- and postintervention impact on self-reported outcomes and implementation, and lay and professional views	To investigate pre- and postintervention impact on self-reported outcomes and implementation, and lay and professional views	To investigate pre- and postintervention impact on self-reported outcomes and implementation, and lay and professional views
Number of participants (n)	Preschool children (1500) Parents (20) Staff (30) Stakeholders (5)	Parents (200) Facilitators (10) Stakeholders (5)	Parents (140) Facilitators (10) Stakeholders (5)	Parents (100) Facilitators (12) Stakeholders (5)
Data type	Early years outcomes, well-being scales, interviews	Self-report measures and interviews	Self-report measures, parenting questionnaires, and interviews	Self-report measures, parenting questionnaires, and interviews

^a^HENRY: Healthy Exercise Nutrition for the Really Young.

These projects already have systems in place to collect outcome data pre- and postintervention, with some opportunities to compare outcome data with existing larger datasets. Two of the four chosen are established programs running elsewhere in the United Kingdom or internationally, with published evidence demonstrating improved outcomes.

In terms of conducting evaluation research in Luton, we identified further cross-cutting questions resulting from the development of an in-depth knowledge of both provision and context, such as how established programs perform when implemented in highly ethnically and culturally diverse populations and the extent to which these complex interventions can be tailored to local circumstances or allow a degree of adaptation [2]; whether Flying Start is able to replicate the positive results reported from pilot studies or improve on outcomes published elsewhere; and collecting qualitative process data to understand *how* such improved outcomes were achieved (or not), which is an aspect of particular value to Flying Start, given that projects initially tend to be rolled out on a small scale.

Consequently, an overarching process-evaluation model was developed, which could be applied and tailored, where appropriate, to all the projects to be evaluated:

- Observations of staff and facilitator training sessions
- Observations of intervention sessions with facilitators working with families and children
- Individual interviews (or focus groups, where deemed appropriate) with staff once interventions are running as well as with project leads and Flying Start leads/commissioners
- Interviews with families after completing the sessions with follow-up interviews at 6 and 12 months

The five steps described then lead to the sixth step, developing individual evaluation protocols.

### Step 6 – Negotiating With Chosen Project Leads Both Within and Outside the Organization

In order to develop a separate, specific evaluation protocol per project, it was necessary to liaise with project leads from within and outside Flying Start. From an academic point of view, the protocols were intended to stand alone as a plan to conduct a robust process and outcome evaluation; however, it was vital to receive regular feedback from Flying Start staff on what was achievable. This would include negotiating access to observe particular staff training sessions, meetings, mentoring, and shadowing routine visits and key program sessions with families; quantifying as precisely as possible the level of involvement required from all stakeholders named in each evaluation to allay the anxieties of stressed staff with challenging workloads; and actively listening to personnel involved at all levels about their concerns and aspects/dimensions of the programs that they particularly wanted to know more about, given their expertise of the local context and population demographics. While working in detail to map out the evaluation stages required for each intervention, it was necessary to remain aware of the organization’s provision as a whole, specifically, the potential themes underlying all projects delivered.

In the case of Flying Start provision, a training course attended by a large proportion of Luton’s early years workforce—Five to Thrive [40,41]—was of particular interest; this course coaches staff to apply evidence-based neuroscientific approaches in their practice to support families to strengthen attachment bonds by responding, talking, playing, relaxing, and cuddling their children. Representing a cornerstone of the Flying Start strategy, all study protocols included the aim of investigating the impact of this training on staff as a part of each process evaluation.

### Step 7 – Development of Individual Project Evaluation Protocols

Protocol drafts were revised on numerous occasions, as Flying Start staff commented and questioned the evaluation approach and content. For particular projects where Flying Start had subcontracted part of the delivery to a partner organization offering the intervention, the protocols were also sent out externally for feedback and clarification as well as to academic colleagues based in other universities with prior/continuing involvement with the development of the original intervention or evaluations thereof. Once protocol drafts were approved, topic guide questions were formulated for the proposed process evaluation for each project. These questions were tailored for interviews with different stakeholders—Flying Start leads/commissioners, project leads, frontline staff/session facilitators, and families. Again, the draft questions were circulated to all Flying Start staff (and key external program staff, where appropriate) involved in the delivery of each project in order to draw upon their expertise and ensure key topics were explored sufficiently and no aspects were overlooked. With agreement on the content of the topic guides, information sheets, and consent forms, it was possible to apply for ethical approval.

### Step 8 – Applying for Ethical Approval From the University and Partner Organization

Although the National Health Service in the United Kingdom, for example, has systems in place as a result of systematic and ongoing evaluations of health interventions, a local authority working with an academic partner is less common. Ethical approval was sought from both the University of Bedfordshire and Luton Borough Council. Applications had to fulfil requirements of both the Institute for Health Research and Luton Borough Council for participant informed consent, data protection, and data storage. We worked with the Council’s Information Governance Team to ensure the requirements of their Tier 3 Information Sharing Agreement, detailing data-sharing processes for each Flying Start project under evaluation, were met as well as to write a master Information Sharing Agreement outlining the overarching principles all parties must adhere to as part of the evaluation research process. Additional time was required to ensure that the Council’s data-sharing and informed consent guidance was met, which must also adhere to European Union law in this area. Finally, each research protocol was registered in the ISRCTN (International Standard Registered Clinical/soCial sTudy Number) registry to maximize awareness of the evaluations to other researchers, clinicians, and the public as well as to promote transparency and reduce duplication and selective reporting [27,42-44].

## Discussion

### Overview

This paper describes the preparation and work required to identify multiple complex projects/interventions suitable for process and outcome evaluation from a range of provision offered by an organization (in this case, services), designed to improve early years outcomes as part of local council provision in the United Kingdom. It details the complexities of academic partners working with a local authority to lay the foundation for a robust evaluation, with the aim of sharing this learning with others who are considering working within a similar model. We outline these steps in relation to previous guidance on conducting evaluations and the preassessment of specific interventions, namely, evaluability assessment, prior to embarking on evaluation research.

Our work adds to existing literature on evaluation methodology by setting out the steps required, particularly related to academic partners and organizations tasked with delivering services designed to meet local needs. After the 8 steps are completed, or the process is in the latter stages, a detailed evaluability assessment may be carried out. This may be particularly important if the projects identified are large scale, costly interventions requiring considerable resources to evaluate and pressure to produce conclusive results. Furthermore, an advantage of using the 8 steps prior to evaluability assessment is that many of the questions about an intervention’s performance and expected outcomes have already been explored before a more detailed appraisal can be made about intervention management and performance. For smaller scale interventions, the use of the 8-Step Scoping Framework may be sufficient to allow progression to the evaluation-planning stage.

### Limitations

The 8-Step Pre-evaluation Framework covers the early stages of evaluation planning to identify complex interventions suitable for evaluation. Therefore, this paper does not address economic aspects such as a cost-benefit analysis of late intervention [45,46] and how including such expertise may strengthen an evaluation and offer a business case for future funding or commissioning decisions [47]. Further work should be undertaken to address how and when economic expertise would fit in to evaluation planning and how additional resources would be factored in to allow for this.

The 8 steps described are tested in relation to academic institutions working with local government in order to build an evidence base but is intended to be applied in other contexts where the goal is to develop a program of evaluation to identify what works. This could be, for example, healthcare, national government, or educational settings. Key criteria are that some form of program, provision, or service be offered with a defined purpose to change or improve a particular outcome(s). Therefore, at this stage, it could be argued that the 8-Step Scoping Framework may be applied in a wide variety of settings and contexts where academic evaluation is required. However, further refinement will likely be required, as others apply the 8-Step Framework and report on its generalizability and their experiences of identifying projects/interventions for evaluation.

The 8 steps are contingent upon an organization being open to having their provision evaluated and to change or modify procedures to ensure that data collection can take place. It requires researchers with good interpersonal and communication skills who are able to ask the pertinent questions and develop positive working relationships [47]. It is also important to note that the process of identifying suitable interventions is, in part, iterative and dependent on context, with some stages overlapping and feeding into each other in order to maintain momentum and ensure the most efficient use of time while considering a wide range of provision.

### Evaluation Challenges, and Future Plans

The 8-Step Scoping Framework will be refined by continuing to work with Flying Start to identify further projects for evaluation in 2019 as well as seeking detailed feedback on how the organization has found the experience of working with an academic partner and being the subject of evaluation activities. As the evaluation of the chosen projects progresses, it will be possible to reflect further and refine the steps set out in this paper. The ongoing impact of evaluation work is a dynamic process. As early results emerge, positive effects will reinforce original decisions to build an evidence base, whereas less conclusive or negative results may lead to skepticism and disappointment. Considering the weight of expectations around evaluation, a regular dialogue about the impact of results, coupled with a reminder of how process findings should help improve different aspects of provision, may help resolve any arising issues. We highlight the importance of developing positive working relationships and harnessing the expertise of organizations to ensure an evaluation asks the pertinent questions and explores the key issues.

Four Flying Start projects were found to be suitable for evaluation: Sign 4, HENRY, Incredible Years, and Parents as Partners. The selected projects fulfil our evaluation criteria to varying degrees: They collect key outcome data, allow comparisons with control groups, are established or imminent, are sizeable and scalable to allow for a mixed-methods approach, and use databases to allow tracking over time and have scope for inclusion of follow-ups. Our process and outcome-evaluation framework will enable us to assess what works and why it works. The steps identified have been described in the context of early years provision but can be applied to broader community-based evaluations. The process of formulating key evaluation questions in step 1 will ensure that the overarching evaluation strategy will retain its focus and we continue to be aligned with the objectives the organization.

Our subsequent evaluation of Flying Start provision must set realistic and achievable goals with the help of a detailed timetable, including contingency plans and a degree of slippage. Consideration must be given to issues of fidelity, whereby interventions may differ substantially between areas/settings, as projects need to adapt and take into account the needs of different communities. The evaluations must also consider difficult-to-reach groups who are less likely to access Flying Start services and that in the process of working with diverse communities, people do not fit into nicely packaged intervention and evaluation “boxes”.

## Appendix

Multimedia Appendix 1 Data template for the project/program offered.

## Abbreviations

HENRY: Healthy Exercise Nutrition for the Really Young
LBC: Luton Borough Council
