# Supplementary material for: Identification of Complex Health Interventions Suitable for Evaluation: Development and Validation of the 8-Step Scoping Framework
Source: JMIR Res Protoc. 2019 Mar 5;8(3):e10075. doi: 10.2196/10075 (PMC6423464; doi:10.2196/10075)
Supplement: Multimedia Appendix 1 [file resprot_v8i3e10075_app1.pdf]

**Name of intervention**	
**Name of lead**	
**Brief description**	
**Target audience- please provide details**<br>i.e.<br>• Pregnant women / partner<br>• Family with child/ren<br>• Age of child/ren<br>• Parents of the future<br>• Specific groups with specific characteristics?	
**Start date**	
**Did you collect any baseline data before starting the programme/intervention?**	
**Length of intervention?**<br><br>***Is this a pilot?***	
**What are the key aims of the intervention?**	
**The intervention links with which Flying Start overarching outcomes?**	Communication and language<br><br>Healthy pregnancy and nutrition and diet<br><br>Social and emotional<br><br>Systems including workforce, volunteering
**What data is available to demonstrate impact? Routine data, validated tools,**	

process data?				
	What data is available and what does this tell us?	Who owns the data? Please state if in public domain or locally held data intelligence	How often is it reported?	Who is custodian of the data?
**Key KPI's short term**				
**Key KPI's medium term**				
**Key KPI's longer term**				
**What opportunities are there for tracking cohorts?**<br><br>***What tools would be needed?***				
**Would interviews/ focus groups/ observations be possible as part of evaluation of intervention?**				
**How long would you estimate before we can expect to see**				

**change?**	
**What is the size and scale of the intervention – how many people with the intervention be working with?**	
**What it the coverage of intervention?**<br>• **town wide**<br>• **Ward focused? If so which ward/s**	
**Are participants likely to be involved in more than one programme? If so, please describe**	
**Other comments**	
